# A learning-based method to predict LncRNA-disease associations by combining CNN and ELM

**DOI:** 10.1186/s12859-022-04611-3

**Published:** 2022-03-22

**Authors:** Zhen-Hao Guo, Zhan-Heng Chen, Zhu-Hong You, Yan-Bin Wang, Hai-Cheng Yi, Mei-Neng Wang

**Affiliations:** 1 School of Electronics and Information Engineering, Tongji University, No. 4800 Cao’an Road, Shanghai, 201804 China; 2 College of Computer Science and Engineering, Shenzhen University, Shenzhen, 518060 China; 3 School of Computer Science, Northwestern Polytechnical University, Xi’an, 710129 China; 4 College of Information Science and Engineering, Zaozhuang University, Zaozhuang, 277100 Shandong China; 5 Xinjiang Technical Institute of Physics and Chemistry, Chinese Academy of Sciences, Urumqi, 830011 China; 6 University of Chinese Academy of Sciences, Beijing, 100049 China; 7 School of Mathematics and Computer Science, Yichun University, Yichun, 336000 Jiangxi China

**Keywords:** CNN, ELM, lncRNA, Disease, Association prediction

## Abstract

**Background:**

lncRNAs play a critical role in numerous biological processes and life activities, especially diseases. Considering that traditional wet experiments for identifying uncovered lncRNA-disease associations is limited in terms of time consumption and labor cost. It is imperative to construct reliable and efficient computational models as addition for practice. Deep learning technologies have been proved to make impressive contributions in many areas, but the feasibility of it in bioinformatics has not been adequately verified.

**Results:**

In this paper, a machine learning-based model called LDACE was proposed to predict potential lncRNA-disease associations by combining Extreme Learning Machine (ELM) and Convolutional Neural Network (CNN). Specifically, the representation vectors are constructed by integrating multiple types of biology information including functional similarity and semantic similarity. Then, CNN is applied to mine both local and global features. Finally, ELM is chosen to carry out the prediction task to detect the potential lncRNA-disease associations. The proposed method achieved remarkable Area Under Receiver Operating Characteristic Curve of 0.9086 in Leave-one-out cross-validation and 0.8994 in fivefold cross-validation, respectively. In addition, 2 kinds of case studies based on lung cancer and endometrial cancer indicate the robustness and efficiency of LDACE even in a real environment.

**Conclusions:**

Substantial results demonstrated that the proposed model is expected to be an auxiliary tool to guide and assist biomedical research, and the close integration of deep learning and biology big data will provide life sciences with novel insights.

## Background

In the past few decades, it is believed that only the protein-coding genes contain genetic information [1]. As the development continues to deepen, researchers found that the number of noncoding RNAs (ncRNAs) in the whole transcriptome is over 98% [2], which makes it confident to believe that ncRNAs may be a kind of biomolecules with abundant functions [3–5].

Long non-coding RNA (LncRNA) is a kind of ncRNA of which length longer than 200 nucleotides [6]. At first, the low expression level and high tissue-specific pattern of lncRNA mislead many researchers to treat it as “transcriptional noise”. Accumulated studies have proved that lncRNA is involved in many life activities such as immune system, genome regulation, and cell-fate programming and reprogramming [7]. There is also a great number of researches confirm numerous human diseases such as cancers, blood diseases and neurodegeneration are associated with various kinds of lncRNAs [8]. Therefore, it is critical and urgent to identify uncovered human lncRNA-disease associations to facilitate understanding the mechanisms [9–11].

It is unrealistic to confirm uncovered lncRNA-disease associations by large-scale wet experiments in terms of time consumption, high cost and high error rate [12]. Significant advances achieved by Artificial Intelligence (AI) and computational methods have had a huge impact in a wide field [13–16]. Due to the assumptions that similar lncRNAs are associated with similar diseases and vice versa [17]. Computational methods for the detection of uncovered relationships have become a hot topic in bioinformatics [18, 19] based on some related databases such as MNDR [20], Lnc2Cancer [21], NONCODE [22] and DrugBank [23].

To date, there are approximately 3 categories of methods for predicting potential associations or interactions between different bioentities. The first kind of methods is based on the matrix decomposition. Lu et al. [24] proposed a method called SIMCLDA to predict the lncRNA-disease potential association based on the induction matrix by combining ontology associations and function similarity. Chen et al. [25] present a novel framework called IMCMDA to infer potential miRNA-disease associations. Secondly, a large number of computational models predict associations borrow the idea of network. Chen et al. [26] propose a computational method to discover unknown drug-target interactions by network-based random walk with restart. Zhou et al. [27] proposed a rank-based method called RWRHLD to predict lncRNA-disease association by prioritizing candidate lncRNA-disease integrated networks. Thirdly, machine-learning-based methods for detecting disease-related miRNAs have been extensively mined. Guo et al. [28] proposed a supervised machine learning method based on various biological information. Computational methods could obtain new lncRNA-disease associations in a short time, which significantly provides a broad prospect for low-risk and faster medical development [29]. The combination of control theory, machine learning and big data will provide relevant researchers with novel insights [30–33].

From the collection of data to the construction of computational models, lncRNA has attracted a lot of attention in the field of computational biology [34–36]. Chen et al. [37] developed a database called ncRNA Drug Targets Database (NRDTD) that collected clinically or experimentally supported ncRNAs as drug targets. Sun et al. [38] constructed a database called Disease Related LncRNA-EF Interaction Database (DLREFD), which contains experimentally verified interactions among lncRNAs. Liu et al. [39] proposed a computational model to infer lncRNA-disease associations by combining human lncRNA expression profiles, gene expression profiles, and human disease-associated gene data.

**Fig. 1:**
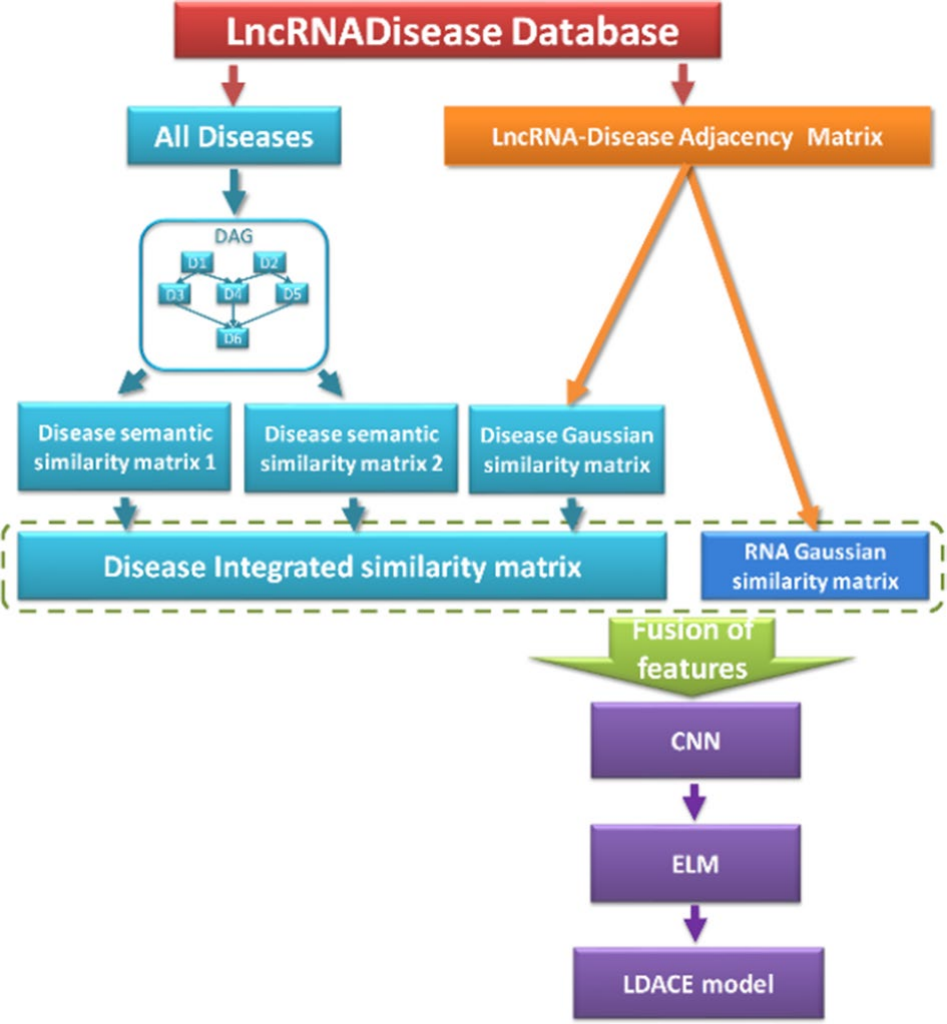
Flowchart of the proposed model LDACE

In this paper, we proposed a novel learning-based prediction model called LDACE by combining CNN and ELM. The framework of the proposed method can be seen in the Fig. 1. Firstly, we downloaded known lncRNA-disease associations from LncRNADisease database [40] in October, 2018. 1765 independent associations consist of 328 different diseases and 881 different lncRNAs were obtained after removing redundant and invalid items. Then, an adjacency matrix could be constructed with above data to store the whole information. Secondly, the semantics similarity matrix and Gaussian interaction profile kernel similarity matrix of disease or lncRNA are calculated respectively to enable lncRNA or disease to be represented by abundant biological information. Finally, after feature selection and dimension transformation by CNN, the low-dimension vectors in a suitable space are taken into the ELM classifier for training, validation and test. As a result, LDACE obtained substantial performance with Area Under Receiver Operating Characteristic Curve (AUROC) of 0.9057 under Leave-one-out cross-validation (LOOCV) and 0.8994 under fivefold cross validation. Moreover, the classifier and method comparison experiments are applied to assess the ability of the proposed model from different aspects. In addition, we also carried out 2 kinds of case studies to simulate the prediction effect of LDACE in the real environment. Considering the competitive performance of the various results under numerous evaluation criteria implemented, the proposed method can indeed serve as a guidance for practice. Meanwhile, this work can be viewed as a attempt to combine machine learning method with biological big data. It is anticipated to provide novel insight to understand mechanism and cell activity at molecular level for related biomedical researchers.

## Results and discussion

### Evaluation criteria

Cross validation was chosen to carry out the evaluation task to assess the performance fairly and comprehensively. For k-fold cross-validation, the whole data set is divided into k mutually exclusive subsets of equal size, each subset can be treated as the test set to evaluate the model in turn, and the others are utilized as the training set to construct the model. When cross-validation is implemented, ROC and AUROC are drawn and calculated separately. ROC can be used at different thresholds to evaluate the ability of the model. The area of the ROC is Area Under the Curve (AUROC). When the AUROC is equal to 1, the classifier will generate a perfect prediction result. If the AUROC value is 0.5, this classifier can be treated as a random guess. A wide range of evaluation methods are used to assess our methods in a different way including accuracy (Acc.), sensitivity (Sen.), specificity (Spec.), precision (Prec.), and MCC. They are defined as:

1 $$Acc. = \frac{TN + TP}{{TN + TP + FN + FP}}$$

2 $$Sen. = \frac{TP}{{TP + FN}}$$

3 $$Spec. = \frac{TN}{{TN + FP}}$$

4 $$Prec. = \frac{TP}{{TP + FP}}$$

5 $$MCC = \frac{TP \times TN - FP \times FN}{{\sqrt {\left( {TP + FP} \right)\left( {TP + FN} \right)\left( {TN + FP} \right)\left( {TN + FN} \right)} }}$$

where *TP* denotes the number of true positives; *FP* represents the number of false positives; *TN* indicates the number of true negatives; *FN* stands for the number of false negatives.

### Leave-one-out cross validation (LOOCV)

**Fig. 2:**
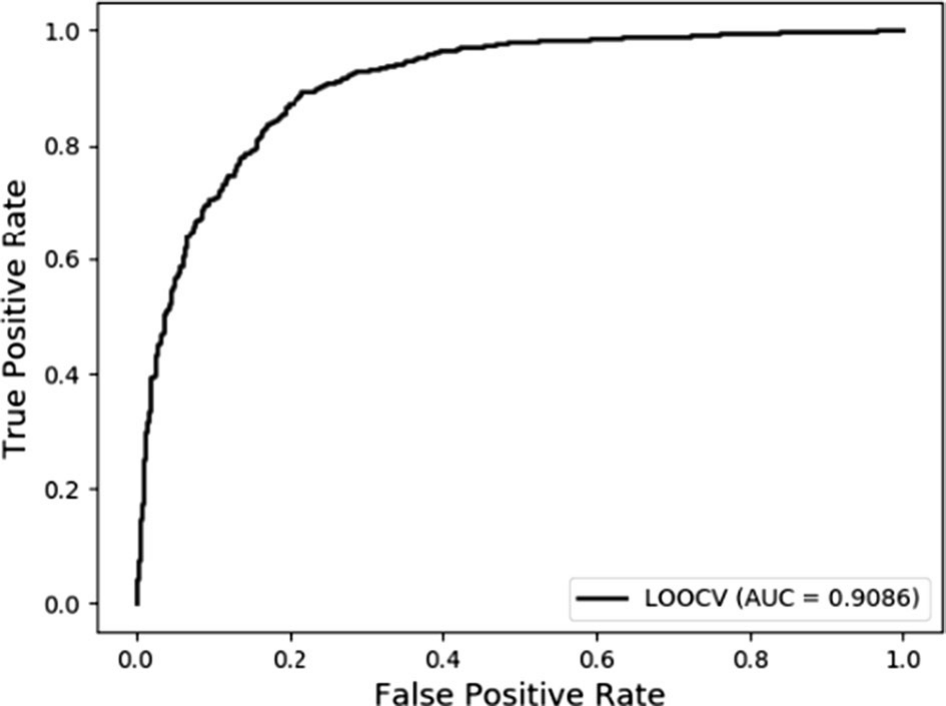
The ROC and AUROC achieved by LDACE in LOOCV on v2018 dataset (3530 lncRNA-disease associations)

For Leave-One-Out Cross Validation (LOOCV), only one sample is left as the test set at each time, and the others are treated as the training set to build the model. The total number of the whole v2018 dataset is 3530, so we repeat 3530 times to train and test in the end. For LOOCV, LDACE obtained a competitive AUROC of 0.9086. The ROC and AUROC achieved by the proposed method can be seen in Fig. 2.

### Fivefold cross validation

**Fig. 3:**
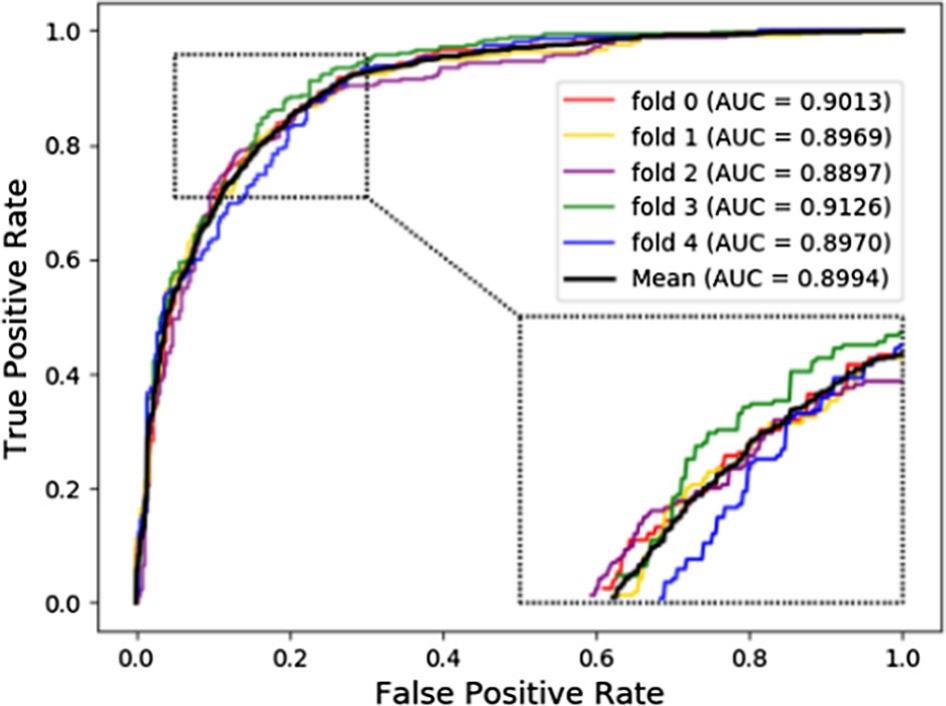
ROCs and AUROCs achieved by LDACE under fivefold cross validation on the v2018 dataset

Considering that LOOCV is labor-intensive, time-consuming and limited by real-world experiment. Fivefold cross validation was chosen to evaluate the proposed model from another perspective. As described in the above section for the k-fold cross validation, it is required to repeat 5 times under this kind of evaluation strategy to obtain the final predictive performance. Specifically, LDACE achieved mean AUROC of 89.94% under fivefold cross validation with a 0.84% standard deviation. A various of evaluation metric including Acc., Sen., Spec., Prec. and MCC were 82.52%, 85.04%, 80, 81%, 65.19% and 89.95%, respectively. Their standard deviations were 0.61, 2.76, 2.12, 1.19 and 1.33. The high AUROC obtained by LDACE implied that the proposed model with various types of biological information indeed was reliable and effective to discover the potential lncRNA-disease associations. The low standard deviation demonstrated that LDACE was stable and robust. The results of the proposed method can be seen in Table 1 and Fig. 3.

**Table 1:** Various evaluation criteria under fivefold cross validation achieved by LDACE on v2018 dataset

Fold	Acc. (%)	Sen. (%)	Spec. (%)	Prec. (%)	MCC (%)	AUROC (%)
0	82.29	83.00	81.59	81.84	64.60	90.13
1	82.44	82.44	82.44	82.44	64.87	89.69
2	82.01	83.85	80.17	80.87	64.07	88.97
3	83.57	88.67	78.47	80.46	67.49	91.26
4	82.29	87.25	77.34	79.38	64.91	89.70
Average	82.52 ± 0.61	85.04 ± 2.76	80.00 ± 2.12	81.00 ± 1.19	65.19 ± 1.33	89.95 ± 0.84

### Classifiers comparison

**Fig. 4:**
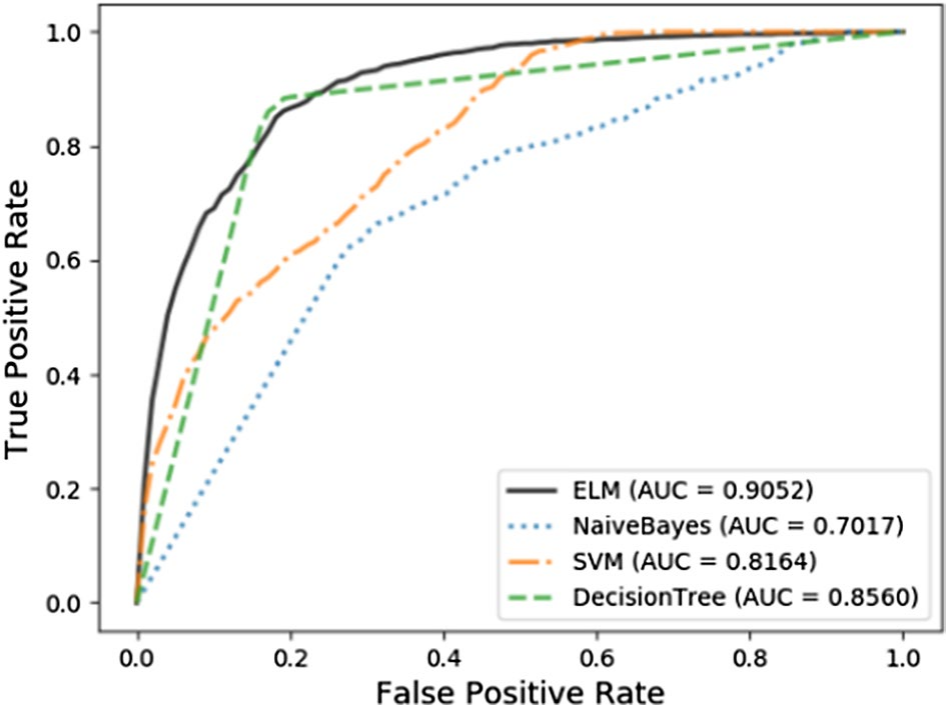
Classifiers comparison under fivefold cross validation on the v2018 dataset

In order to evaluate the performance of ELM in this dataset, we compared ELM with some commonly used classifiers in this section. Under fivefold cross validation, the ROC and the AUROCs are as in the Fig. 4. For fairness, all parameters are set to default values and it is obvious that ELM achieved the most competitive results. The effective ability of ELM can be attributed to the following factors: (1) For NaïveBayes, each feature of the representation vector may not be independent which makes the classification effect dissatisfied. (2) For SVM, training and test samples may be linearly inseparable, and the choice of kernel function under default parameters is not optimal. (3) For decision tree, it is easy to over fit and ignore the correlation between attributes. ELMs with fewer training parameters, faster speeds, and a wide range of applications is chosen to perform the final classification task.

### Compared with previous methods

To further assess the performance of our method with existed methods, LDACE was compared with other 3 network-based models including LRLSLDA [41], LRLSLDA-LNCSIM1 [42] and LRLSLDA-LNCSIM2 [42]. Considering previous model was implemented on the previous dataset which was collected from LncRNADisease in October, 2012. For the sake of fairness, we also applied the proposed framework to train, validate and test on the same version 2012 dataset. The ROC and AUROC obtained by the LDACE can be seen in the Table 2. In conclusion, the proposed computational method increases the AUROC of 0.08, 0.043, and 0.0362, respectively. In addition, machine learning-based models have significant advantages when dealing with new sample problems compared to network-based models.

**Table 2:** The comparison of AUROC between the proposed model and several previous network-based methods in LOOCV on the v2012 dataset

Method	LDACE	LDARF	LRLSLDA-LNCSIM1	LRLSLDA-LNCSIM2
AUROC	0.8560	0.7760	0.8130	0.8198

As a result, the proposed method increases the AUROC of 0.08, 0.043, and 0.0362

### Case study

To further have a more comprehensive evaluation of the proposed model in the real world, we implemented LDACE on lung adenocarcinoma and endometrial cancer as 2 kinds of case studies. The associations in the LncRNA Disease were treated as the training set to construct the computational model, and the other 3 databases including LncRNADisease 2.0 [43], Lnc2Cancer [21], MNDR [20] and CRlncRNA [44] were utilized to verify the prediction results.

In the first kind of case study, lung adenocarcinoma was selected as the research object. Positive samples are all associations existed in the LncRNADisease database and the number of them is 1765. Negative samples were of the equal size as the positive pairs randomly selected from unlabeled associations as mentioned above. The training set consists of both positive samples and negative samples was together sent to ELM for construction of the prediction model. We combined lung adenocarcinoma with all 881 lncRNAs appeared in LncRNADisease as the test set and sorted the prediction results to conveniently validate in other database. In the end, the probability of H19 was in 4/881 of the list. It has been associated with lung adenocarcinoma by recent researches [45] and it did not include in the LncRNADisease database.

In the second kind of case study, endometrial cancer was selected as the subject. In order to test the ability of the proposed model in solving new sample problems that is the new lncRNA prediction. Positive samples are composed of the remaining associations that do not contain endometrial cancer related pairs in LncRNADisease. Given that there are 48 endometrial cancer associated pairs, the number of positive samples is 1717 (1765 − 48). Like case study 1, we also randomly extracted and built the same number negative and test samples by similar method. After the construction of the classifier, we put the test set into the computational model for prediction and verified them in the other databases. The list of the validated top 10 lncRNAs can be seen as Table 3.

**Table 3:** Top 10 lncRNAs associated with endometrial cancer which were predicted by LDACE

Num	lncRNA	Confirmed database	Degree in the original dataset
1	snhg4	Unconfirmed	1
2	malat1	CRlncRNA/MNDR/LncRNADisease	61
3	hulc	Unconfirmed	13
4	tusc7	Unconfirmed	7
5	ifng-as1	Unconfirmed	2
6	miat	LncRNADisease	11
7	meg3	CRlncRNA/MNDR/LncRNADisease	46
8	hotair	CRlncRNA/MNDR/LncRNADisease	61
9	kcnq1dn	Unconfirmed	2
10	tug1	LncRNADisease	24

We carefully analyzed the model construction process and the predicted ranks. We think that the result is due to the following factors. From the view of model, due to the assumptions that similar lncRNAs are associated with similar diseases and vice versa. lncRNA and disease are mainly represented by known associations. Therefore, nodes with large degrees are more likely to be predicted. On the other hand, miRNA with numerous associations may be a hot spot. Several isolated nodes such as snhg4 may actually be associated to disease but has not been verified by wet experiments.

### Discussion

As a kind of regulatory factor in the human cells, lncRNA has proven to be closely related to many complex diseases. However, considering the tedious and low efficiency of manual experiments, numerous calculation methods have been developed to assist in the identification of lncRNA-disease associations. In this paper, we proposed an efficient method to discover potential lncRNA-disease associations. We constructed and integrated multi-type features including disease semantics feature, disease and lncRNA function feature. CNN was applied to extract low-dimensional abstract information from the above integrated features and ELM was applied to carry out the prediction task. The proposed method has achieved competitive performance in cross-validation, method comparison and case study experiments.

More and more similar methods have been proposed to accelerate the process of experiments and expose the internal connection between lncNRAs and diseases. Most of these methods make use of the inherent properties of biological entities such as semantic similarity and known relationships such as functional similarity. There are also some methods that take account of additional biological entities such as genes or other ncRNAs as bridges to assist prediction. The method proposed in this paper contains the above characteristics to a certain extent but is not complete. In the future, based on the premise of sufficient and reliable data, we will expand a richer heterogeneous attribute network centered on lncRNA and disease to accelerate reasoning and discovery. We hope that the method we propose can not only provide novel insights for similar methods, but also accelerate the research process of related experimenters.

## Conclusions

In this paper, a computational model called LDACE was proposed based on CNN and ELM to infer potential associations between lncRNAs and diseases. Specifically, the representation vectors of both lncRNA and disease can be constructed by various biological information including function and semantics similarity. After implementing feature extraction and dimension transformation from original space by CNN, the low-dimension dense vectors were sent into the ELM for prediction task. LDACE obtained a substantial performance of 0.9086 in LOOCV and 0.9014 in fivefold cross validation, respectively. Moreover, we carried out the classifier and method comparison experiment. The results achieved by LDACE highlighted that it is an interesting attempt to combine CNN with ELM, and the deep learning technology can significantly improve the performance of the model to distinguish unknown associations. In addition, 2 kinds case studies based on lung adenocarcinoma and endometrial cancer demonstrated the effectiveness of LDACE in the practical environment. Competitive results indicate that our method has a prominent ability in mining the hidden associations between lncRNA and disease. It is believed that the tight integration of deep learning with biological data will promote the development of all aspects in both computer and life sciences. We hope that our work will not only provide assistance and guidance for manual experiments, but also to open up a novel sight to mine potential information and promote deep understanding from biological data by machine learning method.

## Methods

### lncRNA-disease associations

Known lncRNA-disease associations were collected from the LncRNADisease database in August 2018. 2947 lncRNA-disease association pairs were in the initial downloaded file. After routine preprocessing operations such as identifier unification and redundancy removal, we got v2018 dataset containing 1765 independent lncRNA-disease associations including 881 lncRNAs and 328 diseases. Then we constructed an adjacency matrix *A* with 328 rows and 881 columns to store all associations of the v2018 dataset. The element *A* (*i, j*) was set to 1 if and only if the *i*th disease and *j*th lncRNA was experimentally validated to be associated.

Randomly selecting negative samples from unlabeled samples is a commonly used down sampling technique for construction dataset and widespread in bioinformatics [46]. Therefore, the same number of negative samples as the positive samples are randomly selected to form the whole data set together with the positive samples. The total number of the training set is 3530 containing 1765 experimental valid positive samples and 1765 negative samples.

To compare with the existed methods, we also downloaded the previous lncRNA-disease associations called v2012 dataset from the first published lncRNA-disease association prediction model [41, 42]. After the same operation as mentioned above, we obtained 293 independent associations composed of 118 lncRNAs and 167 diseases which is the same as described in the original paper.

### Disease MeSH descriptors

Medical Subject Headings (MeSH) is a standard controlled vocabulary which aims at indexing life and medical books and journals. It can be roughly classified into 16 categories, including Health Care [N], Publication Characteristics [V], Geographicals [Z]. We downloaded all MeSH descriptors (headings) from National Library of Medicine (NLM) in August 2018 to construct and measure the semantics similarity between lncRNA and disease.

### Disease semantic similarity matrix 1

**Fig. 5:**
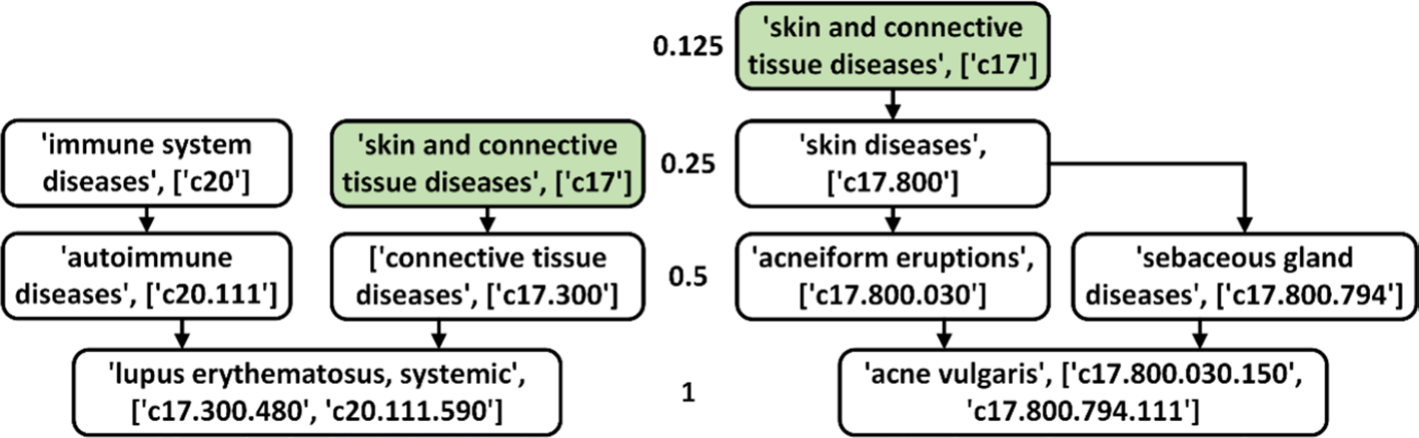
The process of the semantics similarity calculation between diseases “lupus erythematosus, systemic” and “acne vulgaris”. (1) Construct their own directed acyclic graphs according to the rules; (2) Calculate the contribution of various diseases (nodes) to “lupus erythematosus, systemic” and “acne vulgaris” in the directed acyclic graph, of which the lowest level is “lupus” “erythematosus, systemic” and “acne vulgaris” contribute 1 to themselves, and the parent node decays layer by layer, “autoimmune diseases”, “connective tissue diseases”, etc. contribute 0.5, and so on; (3) Calculate “lupus erythematosus, systemic” and “acne vulgaris” constitute the total contribution of directed acyclic graphs. $DV\left( {{\text{lupus erythematosus}},{\text{ systemic}}} \right) = 1 + 2 \times 0.5 + 2 \times 0.25 = 2.5$, $DV\left( {\text{acne vulgaris}} \right) = 1 + 2 \times 0.5 + 1 \times 0.25 + 1 \times 0.125 = 2.375$. $Similarity\left( {{\text{lupus erythematosus}},{\text{systemic}},{\text{acne vulgaris}}} \right) = \frac{0.25 + 0.125}{{2.5 + 2.375}} = 0.0769$

Disease is a kind of abnormal life process that occurs when the body is under certain conditions and is affected by the damage of the disease. How to effectively represent disease as vectors is a difficult task in bioinformatics research for a long period. Previous method has proven that it is a high quality way to characterize disease by MeSH descriptor [47]. The specific calculation step is shown in the Fig. 5. Each disease can be represented as a Directed Acyclic Graph (DAG). For example, disease *D*’s DAG can be represented as *DAG*(*D*) = (*D, *${N}_{D}$*,* ${E}_{D}$), ${N}_{D}$ is a node set which contains disease *D* and its ancestor disease in *DAG*(*D*). ${E}_{D}$ is an edge set which contains all links between nodes in *DAG*(*D*).

Inspired by the Jaccard formula, the similarity can be calculated by dividing the intersection of two sets by the union of two sets. Disease *D* could be represented by a DAG and the semantics similarity between 2 diseases could be calculated as follows:

6 $$\left\{ {\begin{array}{*{20}l} {D1_{D} \left( t \right) = 1 } \hfill & { if\; t = D} \hfill \\ {D1_{D} \left( t \right) = \max \left\{ {\Delta {*}D1_{D} \left( {t^{\prime}} \right)|t^{\prime} \in children{ }of{ }t} \right\}} \hfill & {if\,t \ne D} \hfill \\ \end{array} } \right.$$

where $\Delta$ is the factor and $t$ is the node in *DAG*. $\Delta$ can be from 0 to 1, and it is set to 0.5 according to previous literature [42]. In *DAG (D)*, disease *D* contributes the most to itself. The further the distance is, the smaller the contribution of *D*'s ancestral disease to *D*. Therefore, we can define the sum of the contributions of all nodes in the *DAG(D)* to disease *D*. *DV1*(*D*) can be calculated as:

7 $$DV1\left( D \right) = \Sigma_{{t \in N_{D} }} D1_{D} \left( t \right)$$

The semantic similarity of disease *i* and disease *j* can be defined as follows:

8 $$DS1\left( {i,j} \right) = \frac{{\mathop \sum \nolimits_{{t \in N_{i} \cap N_{j} }} \left( {D1_{i} \left( t \right) + D1_{j} \left( t \right)} \right)}}{DV1\left( i \right) + DV1\left( j \right)}$$

### Disease semantic similarity matrix 2

In the disease semantic similarity matrix 1, the algorithm only forces on a single object from the local view, but does not consider the difference between diseases from the whole perspective. Some scholars believe that the contribution is different because of the appearance frequency of disease in the whole MeSH. Combined with the view of information theory, they proposed novel ideas to improve this situation and achieved a certain degree of improvement [42]. The new contribution of disease *t* to disease *D* can be calculated as follows:

9 $$D2_{D} \left( t \right) = - {\text{log}}\left( {\frac{the number of DAGs including t}{{the number of disease}}} \right)$$

Then the semantic value of disease *D* can be obtained, *DV2(D)* as:

10 $$DV2\left( {\text{D}} \right) = \Sigma_{{t \in N_{D} }} D2_{D} \left( t \right)$$

The semantic similarity of disease *i* and disease *j* can be defined as follows:

11 $$DS2\left( {i,j} \right) = \frac{{\mathop \sum \nolimits_{{t \in N_{i} \cap N_{j} }} \left( {D2_{i} \left( t \right) + D2_{j} \left( t \right)} \right)}}{DV2\left( i \right) + DV2\left( j \right)}$$

### Disease Gaussian interaction profile kernel similarity matrix

Obviously, the matrix *A* includes the whole association contents of the v2018 database. Disease *i* can be represented as a function vector ${\text{d}}_{i}$ of 881 dimensions that is a column of matrix *A*. The value of each dimension in ${\text{d}}_{i}$ is determined by whether disease have been associated with lncRNA or not. If and only if the *i*th disease is valid proved to be associated with the *j*th lncRNA by wet experiment, the *j*th dimension of the vector is defined as 1, otherwise 0.

In fact, this can be treated as a functional representation of the lncRNA, and we transform it by Gaussian interaction profile kernel function to make more suitable for downstream classification tasks. Then similarity between diseases *i* and disease *j* can be defined as follows:

12 $$DG\left( {i,j} \right) = exp\left( { - \alpha_{d} {\text{d}}_{i} - {\text{d}}_{j}^{2} } \right)$$

hyperparameters $\alpha_{d}$ can be defined as follows:

13 $$\alpha_{d} = \alpha_{d}^{^{\prime}} \left( {\frac{1}{nd}\mathop \sum \limits_{i = 1}^{nd} {\text{d}}_{i}^{2} } \right)$$

Here we set $\alpha_{d}^{^{\prime}}$ = 0.5, *nd* is set to 328 which equals to the number of disease. Finally, the disease Gaussian interaction profile kernel similarity matrix *DG* is a square matrix with 328 rows and columns.

### Disease integrated similarity matrix

To integrate all biological information, the element of the final disease similarity matrix *DS* (*i, j*) can be defined as follows:

14 $$DS\left( {i,j} \right) = \left\{ {\begin{array}{*{20}c} {\frac{{DS1\left( {i,j} \right) + DS2\left( {i,j} \right)}}{2} if i and j have semantic similarity } \\ {DG\left( {i,j} \right) otherwise } \\ \end{array} } \right.$$

### LncRNA Gaussian interaction profile kernel similarity matrix

It can represent each lncRNA’s function by the row of the matrix *A* similar to disease. The Gaussian profile kernel similarity between lncRNA *i* and *j* could be calculated as follows:

15 $$RS\left( {i,j} \right) = RG\left( {i,j} \right) = exp\left( { - \alpha_{r} r_{i} - r_{j}^{2} } \right)$$

Given that there is no other information about lncRNAs, we directly regard *RG* as the lncRNA similarity matrix (*RS*). Parameter $\alpha_{r}$ can be adjusted as follows:

16 $$\alpha_{r} = \alpha_{r}^{^{\prime}} \left( {\frac{1}{nr}\mathop \sum \limits_{i = 1}^{nr} r_{i}^{2} } \right)$$

Here, we set $\alpha_{r}^{^{\prime}}$ = 0.5, and *nl* is set to 881 which equals to the number of lncRNA. Finally, the lncRNA similarity matrix *RS* of 328 rows and 328 columns can be constructed.

### The representation of the association pair

From above operations, each lncRNA and disease can be represented as a vector by integrating various biology information. In summary, the *i*th disease can be represented as the *i*th row of the matrix *DS* as shown below:

17 $$DS_{i,*} = \left( {RS_{i,1} ,RS_{i,2} , \ldots ,RS_{i,881} } \right)$$

The *j*th lncRNA can be represented as the *j*th row of the matrix *RS* as shown below:

18 $$RS_{j,*} = \left( {RS_{j,1} ,RS_{j,2} , \ldots ,RS_{j,328} } \right)$$

The combination of the associations between the ith disease and the jth lncRNA is seen as follows:

19 $$AssociationPair_{i,j} = \left( {DS_{i,*} ,RS_{j,*} } \right) = \left( {RS_{i,1} ,RS_{i,2} , \ldots ,RS_{i,881} ,RS_{j,1} ,RS_{j,2} , \ldots ,RS_{j,328} } \right)$$

Then we get 3530 1209-dimensional vectors. Each positive sample is given a label 1 and each negative sample is given a label 0.

### Convolutional neural networks (CNN)

Considering that the constructed representation vector is high-dimensional and sparse, we hope to extract the effective features through Convolutional Neural Network (CNN) [48–50]. Compared to other machine learning method, CNN has its unique advantages in feature capture and model capacity [51]. In this paper, we choose CNN to carry out the feature extraction task [52, 53].

**Fig. 6:**
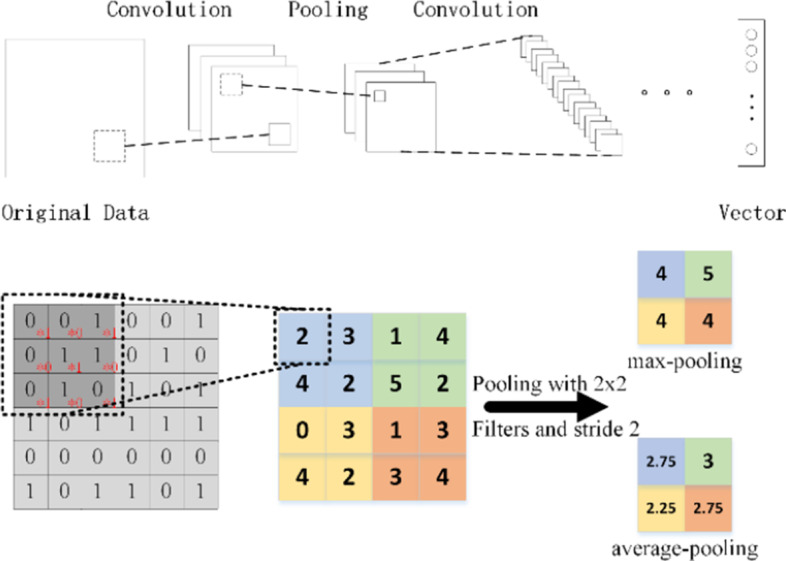
The convolution and pooling of CNN

Convolution neural network is a multi-layer neural network which consists of input layer, convolution layer, pooling layer, fully-connected layer and output layer [54, 55]. The key of CNN lies in the convolutional layer and the pooling layer which extracted features and passed them into the fully connected layer for classification [56]. The weight of the convolution window is adjusted by the feedback result [57]. The convolution layer is applied to extract both local and global features with different filters. It can be shown in the Fig. 6.

### ELM

**Fig. 7:**
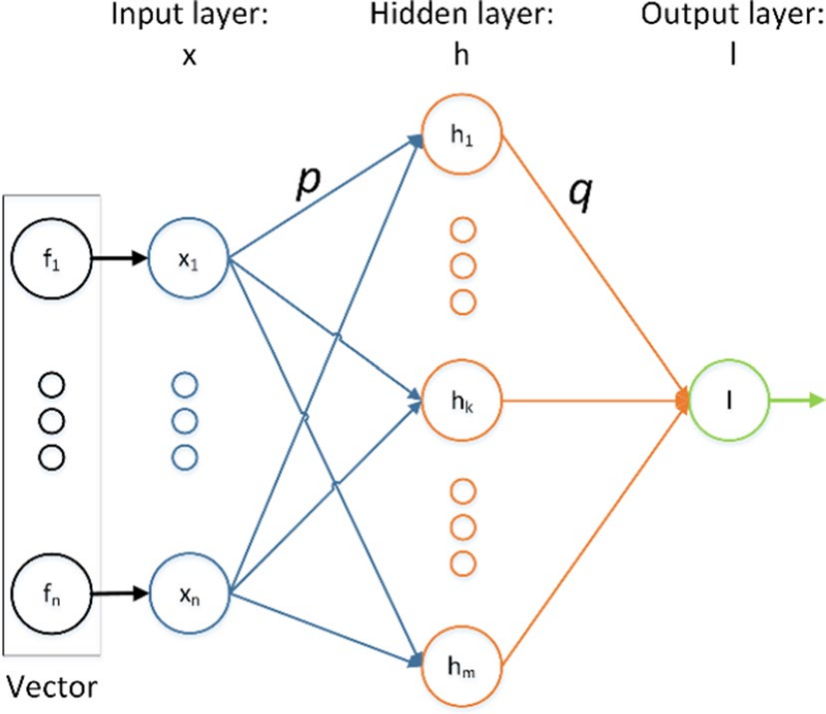
Structure of Extreme Learning Machine (ELM). The connection weight of the input layer and the hidden layer, and the threshold of the hidden layer can be randomly set. The connection weights between the hidden layer and the output layer do not need to be adjusted iteratively, but are determined at once by solving equations

GB Huang et al. [58] proposed Extreme Learning Machine which is a single hidden layer feedforward neural network algorithm. For traditional artificial neural networks, it will consume lots of resources and time to determine the paraments when back-propagation algorithm is applied [59]. Considering these iterative steps, there is only one hidden layer in ELM and when the classifier is trained, the number of hidden layer neuron nodes is the only hyperparameter that has to be set. The main steps of ELM are shown in Fig. 7.

ELM is a kind of single hidden layer feedforward network with random hidden nodes and the activation function *f(x)*. For *N* arbitrary distinct samples $\left( {x_{i} ,l_{i} } \right)$, where $x_{i} = \left[ {x_{i1} ,x_{i2} , \ldots ,x_{im} } \right]^{T} \epsilon R^{n}$ and $l_{i} = \left[ {l_{i1} ,l_{i2} , \ldots ,l_{im} } \right]^{T} \epsilon R^{m}$. Therefore, the output of ELM is represented as follows:

20 $$\mathop \sum \limits_{i = 1}^{{N^{\prime}}} q_{i} f\left( {p_{i} \cdot x_{j} + t_{i} } \right) = O_{j} , j = 1, \ldots ,N$$

where $N^{\prime}$ is the number of the hidden nodes, $p_{i} = \left[ {p_{i1} ,p_{i2} , \ldots ,p_{im} } \right]^{T}$ is the weight vector from the input layer nodes to the *i*th hidden layer node, $q_{i} = \left[ {q_{i1} ,q_{i2} , \ldots ,q_{im} } \right]^{T}$ is the weight vector from the *i*th hidden layer to the output layer, $t_{i}$ is the threshold of the *i*th hidden node. $p_{i} \cdot x_{j}$ is the inner product of $p_{i}$ and $t_{i}$.

The loss function is defined as follows:

21 $$\mathop \sum \limits_{j = 1}^{N} O_{j} - l_{j}$$

In order to minimize the error between input and output, we need to determine the three parameters $p_{i} ,q_{i} and t_{i}$ such that:

22 $$\mathop \sum \limits_{i = 1}^{{N^{\prime}}} q_{i} f\left( {p_{i} \cdot x_{j} + t_{i} } \right) = t_{j} , j = 1, \ldots ,N$$

The Eq. (22) can be written compactly as $Hq = l$ where

23 $$\begin{aligned} & H\left( {p_{1} , \ldots ,p_{{N^{\prime}}} ;q_{1} , \ldots ,q_{{N^{\prime}}} ;X_{1} , \ldots ,X_{N} } \right) \\ & \quad = \left[ {\begin{array}{*{20}c} {g\left( {p_{1} \cdot x_{1} + t_{1} } \right)} & \cdots & {H\left( {p_{{N^{\prime}}} \cdot x_{1} + t_{{N^{\prime}}} } \right)} \\ \vdots & \ddots & \vdots \\ {g\left( {p_{1} \cdot x_{N} + t_{1} } \right)} & \cdots & {H\left( {p_{{N^{\prime}}} \cdot x_{N} + t_{{N^{\prime}}} } \right)} \\ \end{array} } \right]_{{N \times N^{\prime}}} , q = \left[ {\begin{array}{*{20}c} {q_{1}^{T} } \\ \vdots \\ {q_{{N^{\prime}}}^{T} } \\ \end{array} } \right]_{{N^{\prime} \times m}} , l = \left[ {\begin{array}{*{20}c} {l_{1}^{T} } \\ \vdots \\ {l_{N}^{T} } \\ \end{array} } \right]_{N \times m} \\ \end{aligned}$$

Therefore, in order to train the ELM, we need to find the appropriate parameters $\hat{p}_{i} ,\hat{q}_{i} and \hat{t}_{i}$ such that

24 $$H\left( {\hat{p}_{i} ,\hat{t}_{i} } \right)\hat{q}_{i} - l = \mathop {min}\limits_{p,q,t} H\left( {\hat{p}_{i} ,\hat{t}_{i} } \right)\hat{q}_{i} - l, i = 1,2, \ldots ,N^{\prime}$$

It is equivalent to minimize the loss function as follows:

25 $$E = \mathop \sum \limits_{j = 1}^{N} \left( {\mathop \sum \limits_{i = 1}^{P} q_{i} f\left( {p_{i} \cdot x_{j} + t_{i} } \right) - l_{j} } \right)^{2}$$

ELM combined high learning efficiency and strong generalization ability is widely used in solving both academic and industrial issues. Here, all hyperparameters are set to default values.

## Data Availability

LncRNA-disease association dataset can be downloaded from the url: http://www.cuilab.cn/. Disease MeSH descriptors can be downloaded from the url: ftp://nlmpubs.nlm.nih.gov/online/mesh/. The code used or analyzed during this study are available from the corresponding author on reasonable requests.

## Abbreviations

LncRNA: Long non-coding RNA
ELM: Extreme Learning Machine
CNN: Convolutional Neural Network
AUROC: Area Under Receiver Operating Characteristic Curve
LOOCV: Leave-one-out cross-validation
DAG: Directed Acyclic Graph
